# *Sly*miR482e‐3p mediates tomato wilt disease by modulating ethylene response pathway

**DOI:** 10.1111/pbi.13439

**Published:** 2020-07-16

**Authors:** Ying Gao, Si‐Jian Li, Shen‐Wei Zhang, Tao Feng, Zhao‐Yang Zhang, Shu‐Jie Luo, Hui‐Ying Mao, Katherine A. Borkovich, Shou‐Qiang Ouyang

**Affiliations:** ^1^ College of Horticulture and Plant Protection Yangzhou University Yangzhou China; ^2^ Department of Microbiology and Plant Pathology Institute for Integrative Genome Biology University of California Riverside CA USA; ^3^ Joint International Research Laboratory of Agriculture and Agri‐Product Safety of Ministry of Education of China Yangzhou University Yangzhou China; ^4^ Key Laboratory of Plant Functional Genomics of the Ministry of Education/Jiangsu Key Laboratory of Crop Genomics and Molecular Breeding College of Agriculture Yangzhou University Yangzhou China

**Keywords:** microRNA, ethylene signalling pathway, *Fusarium oxysporum*, wilt disease, tomato

*Fusarium oxysporum* f. sp. *Lycopersici*, a necrotrophic pathogen, is a causal agent of tomato wilt disease. Plants have two major sophisticated innate immune systems, Pathogen‐Associated Molecular Pattern (PAMP)‐triggered immunity (PTI) and Effector‐Triggered Immunity (ETI), to perceive and resist pathogen offences (Jones and Dangl, 2006). MicroRNAs (miRNAs) contribute to PTI and ETI by fine‐tuning plant hormones and/or silencing the genes involved in pathogen virulence by regulating the expression of target genes, thereby acting as crucial regulators of the plant immune system (Fei *et al.*, 2016). Many plants produce microRNAs belonging to the miRNA482/2118 superfamily. These miRNAs target R‐genes of the class NBS‐LRR (nucleotide‐binding site‐leucine rich repeat) through recognizing the P‐loop motif in the NBS‐LRR mRNA. Our previous studies showed that *Sly*miR482e‐3p, a members of the miR482/2118 superfamily in tomato, negatively regulated the resistance to *Fusarium oxysporum* f. sp. *lycopersici* (race 2) (*Fol*) by targeting several NBS‐LRR genes (Ouyang *et al.*, 2014). However, the exact mechanism underlying the basic function of *Sly*miR482e‐3p during the response to *Fol* attack needs further exploration. In this study, two near‐isogenic tomato cultivars, Moneymaker (susceptible, *i‐2*/*i‐2*) and Motelle (resistant, *I‐2*/*I‐2*) to *Fol* infection, were recruited (Ouyang *et al.*, 2014).

To characterize the functions of *Sly*miR482e‐3p in response to tomato wilt disease, we generated a CRISPR/Cas9‐related knock‐out mutant lacking the *Sly*miR482e‐3p gene in the susceptible cultivar Moneymaker (Deng *et al.*, 2018). Three regenerated plants, termed as *Sly*miR482e‐3p‐KO‐Line 3, 7 and 11, carried 2‐, 9‐ and 6‐nucleatide deletion in front of the mature miRNA region respectively, were identified (Figure 1a). Compared with the control, the expression levels of *Sly*miR482e‐3p was dramatically reduced by more than 90% in individual transgenic plants (Figure 1b). *Sly*miR482e‐3p has been proved as a negative regulator for several targeted NBS‐LRR genes, including *Soly08g075630* and *Soly08g076000* in tomato (Ouyang *et al.*, 2014). As expected, basal expression levels of both *Soly08g075630* and *Soly08g076000* were increased in all transgenic Moneymaker plants (Figure 1b). Furthermore, no visible difference in major agronomic traits, including leaves, flowers and fruits, were observed in transgenic plants compared with the control (Figure 1c).

**Figure 1:**
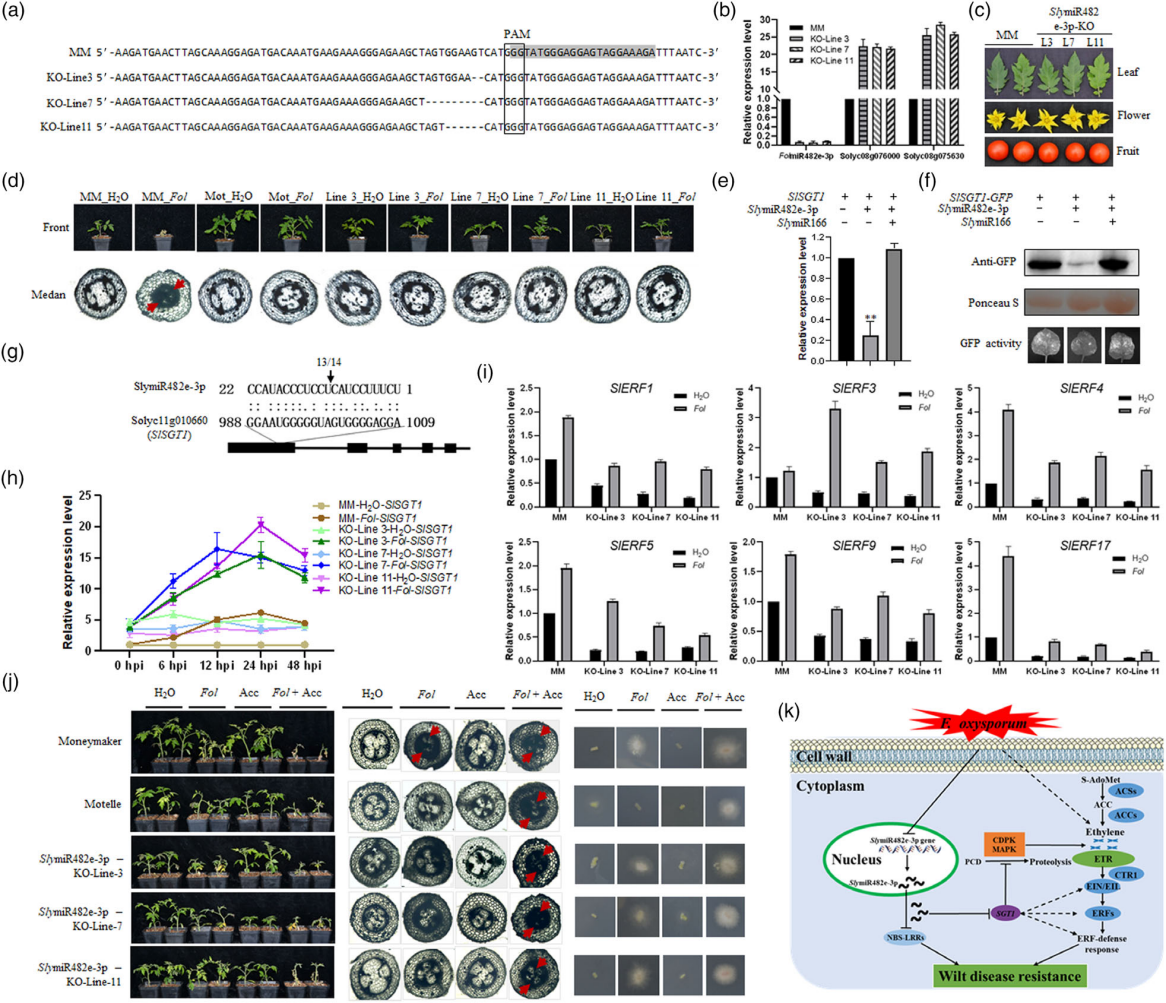
*Sly*miR482e‐3p mediates tomato wilt disease by modulating ethylene response pathway. (a) Clustalx nucleic acid sequence alignments of *Sly*miR482e‐3p‐KO plants. The sequence of mature miRNA is highlighted with grey. (b) Expression of known targets of *Sly*miR482e‐3p in KO plants. (c) Agricultural phenotypic traits of *Sly*miR482e‐3p‐KO plants. (d) Knock‐out of *Sly*miR482e‐3p enhances the resistance to *Fol* in Moneymaker. Two‐week‐old seedlings of the indicated control or transgenic plants were treated with water or *Fol* and photographed 2 weeks later. Red arrows indicating vascular tissue. (e) Level of target mRNAs. qRT‐PCR was used to determine relative levels of *SlSGT1* in *Nicotiana benthamiana* leaves expressing target mRNA and empty vector, target mRNA and the appropriate miRNA, target mRNA and a control miRNA (SlymiR166). Values were normalized to *N. benthamiana* actin. Asterisks indicated significant differences (* *P* < 0.05, ** *P* < 0.01). (f) SlSGT1 protein level was detected by Western blot using anti‐GFP antibody. (g) The cleavage site in the *SlSGT1* mRNA was determined using 5′ RLM‐RACE. The arrow indicates the 5′ terminus of miRNA‐guided cleavage products and the frequency of clones (13/14) was shown. (h) Accumulation of *SlSGT1* during a time course in different cultivars and transgenic tomato plants infected with *Fol*. (i) *Sly*miR482e‐3p regulates ethylene signalling by suppressing the expression of *SlERF*s. Total RNA was isolated from tomato seedling root at 24 hpi and subjected to qRT‐PCR to evaluate expression with gene‐specific primers. (j) Effect of exogenous ethylene on *Fol* defence in wild and *Sly*miR482e‐3p‐KO plants. Two‐week‐old tomato seedlings were inoculated with *Fol* for 30 min followed by the first spraying with 1‐Aminocyclopropane‐1‐carboxylic acid (ACC, 100 μm). All plants were treated three times with an interval of 24 h (Left). Accumulation of *Fol* in tomato stems as visualized by staining with lactic acid phenol Medan dye (Middle). Red arrows indicate vascular tissue boundary of stems with extensive staining. Recovery of *Fol* from tomato stem slices incubated on PDA medium (Right). (k) Model for *Sly*miR482e‐3p‐mediated ethylene signalling in promoting resistance to *Fol*. During fungal pathogen *Fol* invasion, down‐regulated endogenous *Sly*miR482e‐3p releases SlSGT1 accumulation, triggering CDPK‐depending programmed cell death (PCD). Consequently, *SlERF*s, components of the ethylene signalling transduction pathway, are repressed to enhance the resistance to tomato wilt disease caused by *Fol*.

To further evaluate the function of *Sly*miR482e‐3p in tomato wilt disease susceptibility, we inoculated the *Sly*miR482e‐3p‐KO transgenic plants as well as resistant Motelle and susceptible Moneymaker controls with *Fol*. As gauged, *Sly*miR482e‐3p‐KO plants exhibited enhanced resistance to *Fol* relative to the Moneymaker control while displayed an appearance similar to the treated Motelle plants (Figure 1d). This result further confirms that *Sly*miR482e‐3p functions as a negative regulator of resistance to tomato wilt disease.

We utilized the psRNATarget algorithm (Dai *et al.*, 2018) to predict potential targets of *Sly*miR482e‐3p. Intriguingly, *Solyc11g010660*, a homolog of *SGT1* (suppressor of the G2 allele of *skp1*), was predicted as a target of *Sly*miR482e‐3p and termed as *SlSGT1*. SGT1 was first reported as a component of the SCF E3 ubiquitin ligase complex in yeast (Kitagawa *et al.*, 1999) and interacted with RAR1 to trigger disease resistance in plants (Azevedo *et al.*, 2002). It has been documented that SGT1 homologs in plants are triggered by various plant defence response pathways, including ethylene‐mediated cross‐talk between calcium‐dependent protein kinases (CDPK) and mitogen‐activated protein kinase (MAPK) signalling (Ludwig *et al.*, 2005; Peart *et al.*, 2002). To determine whether *Sly*miR482e‐3p regulate the *SlSGT1* expression, we conducted an *Agrobacterium*‐mediated transient co‐expression experiment in *N. benthamiana*, as previously implemented in our laboratory (Ouyang *et al.*, 2014). qRT‐PCR data showed that the *SlSGT1* transcripts were greatly decreased in the presence of *Sly*miR482e‐3p (Figure 1e). Consistently, GFP fluorescence and Western blot assays using an anti‐GFP antibody further demonstrated that SlSGT1 protein levels were significantly down‐regulated in the presence of *Sly*miR482e‐3p (Figure 1f). To identify the cleavage site in the *SlSGT1* mRNA targeted by *Sly*miR482e‐3p, we performed a 5′‐RNA ligase‐mediated rapid amplification of cDNA ends (5′ RLM‐ RACE) analysis. The result showed the cleavage site occurred at the 999th nt of the *SlSGT1* mRNA in 13 out of 14 clones (Figure 1g). *AdSGT1* transcripts were strong up‐regulated by ethephon resulting in enhanced disease resistance in tobacco and peanut (Kumar and Kirti, 2015). In this study, *SlSGT1* was dramatically induced during *Fol* infection in the susceptible cultivar Moneymaker, meanwhile, the basal level of *SlSGT1* was elevated in the *Sly*lmiR482e‐3p‐KO mutant possibly resulting in the resistance to *Fol* (Figure 1h). Considering all of the above results, we concluded that *Sly*miR482e‐3p regulates *SlSGT1* expression by chopping the intact mRNA.

To further understand the role of *Sly*miR482e‐3p in mediating resistance to *Fol* in tomato, we constructed and sequenced six RNA‐seq libraries, including Moneymaker treated with water (MM_H_2_O) or *Fol* (MM_*Fol*), as well as *Sly*miR482e‐3p‐KO lines 3 and 7 treated with water or *Fol* (KO‐Line3_H_2_O, KO‐Line3_*Fol*, KO‐Line7_H_2_O, and KO‐Line7_*Fol*) (The raw sequence data are available in the Genome Sequence Archive in BIG Data Centre, under accession numbers CRA002427). Intriguingly, we determined that genes in several phytohormone signalling pathways, particularly the ethylene (ET) signal transduction pathway, may participate in the response to *Fol* infection in tomato. To evaluate further a possible role for *Sly*miR482e‐3p in regulating ethylene signalling, we monitored expression of key genes in the pathway in tomato plants after inoculation with *Fol* spores or water over a 24 h period. The basal expression levels (water control) of *SlERF1*, *SlERF3*, *SlERF4*, *SlERF5*, *SlERF9,* and *SlERF11* were depressed in all *Sly*miR482e‐3p‐KO plants relative to Moneymaker. However, all these genes except *SlERF3* were induced after *Fol* infection in both Moneymaker and *Sly*miR482e‐3p‐KO plants (Figure 1i). These results prompted us to speculate that the ethylene signal transduction pathway might be important during the response to *Fol* infection. We next asked whether application of a precursor of ET biosynthesis, 1‐Aminocyclopropane‐1‐carboxylic acid (ACC), would exacerbate wilt disease symptoms. For these experiments, WT and transgenic plants were treated with *Fol* followed by spraying 100 μm ACC (optimal concentration was determined through our preliminary experiments). After ACC treatment, all tomato plants displayed aggravated wilt disease symptoms and faster disease progression compared to treatment with *Fol* alone (Figure 1j). Particularly, ACC overrode the resistance to *Fol* infection in Motelle against *Fol* (Figure 1j).

In summary, we present evidence that supports a key role of *Sly*lmiR482e‐3p‐mediated ethylene signalling in promoting resistance to a fungal necrotroph *Fol*. We propose that during fungal pathogen *Fol* invasion, endogenous *Sly*lmiR482e‐3p promotes *SlSGT1* accumulation, thereby triggering CDPK‐depending PCD in tomato. Consequently, *SlERF*s, components of the ethylene signalling pathway are regulated to enhance resistance to tomato wilt disease (Figure 1k). Our research provides a basis to elucidate the complex *Sly*lmiR482e‐3p‐mediated resistance to *Fol* in tomato, which will be beneficial for the design of strategies to improve tomato wilt disease resistance.

## Conflicts of interest

The authors declare no conflict of interest.

## Author contributions

SQO designed the experiments. SQO contributed to data analysis and interpretation and wrote the paper. KAB contributed to design this project and revised this manuscript. YG and SJL performed the experiments in cooperation with SWZ, TF, ZYZ, SJL and HYM. All authors read and approved the final manuscript.
